# Induction of Adipocyte Differentiation by Polybrominated Diphenyl Ethers (PBDEs) in 3T3-L1 Cells

**DOI:** 10.1371/journal.pone.0094583

**Published:** 2014-04-10

**Authors:** Emily W. Y. Tung, Adèle Boudreau, Michael G. Wade, Ella Atlas

**Affiliations:** Environmental Health Science and Research Bureau, Health Canada, Ottawa, Ontario, Canada; University of Barcelona, Faculty of Biology, Spain

## Abstract

Polybrominated diphenyl ethers (PBDEs) are a class of brominated flame retardants that were extensively used in commercial products. PBDEs are ubiquitous environmental contaminants that are both lipophilic and bioaccumulative. Effects of PBDEs on adipogenesis were studied in the 3T3-L1 preadipocyte cell model in the presence and absence of a known adipogenic agent, dexamethasone (DEX). A PBDE mixture designed to mimic body burden of North Americans was tested, in addition to the technical mixture DE-71 and the individual congener BDE-47. The mixture, DE-71, and BDE-47 all induced adipocyte differentiation as assessed by markers for terminal differentiation [fatty acid binding protein 4 (aP2) and perilipin] and lipid accumulation. Characterization of the differentiation process in response to PBDEs indicated that adipogenesis induced by a minimally effective dose of DEX was enhanced by these PBDEs. Moreover, C/EBPα, PPARγ, and LXRα were induced late in the differentiation process. Taken together, these data indicate that adipocyte differentiation is induced by PBDEs; they act in the absence of glucocorticoid and enhance glucocorticoid-mediated adipogenesis.

## Introduction

Polybrominated diphenyl ethers (PBDEs) are a class of brominated flame retardants that were extensively used as additives in commercial products including electronics, textiles, and polyurethane foam [1]. There are approximately 209 congeners of PBDEs that differ based on the degree and position of halogenations [1], [2]. Widely produced commercial PBDE mixtures, including pentabrominated phenyl ether (DE-71) and octabrominated diphenyl ether (DE-79), leach readily into the environment as these chemicals were not chemically bound to the polymers of the products to which they were added [3], [4]. Production of these chemicals was voluntarily halted in 2004; however, concern has been raised for the potential of PBDEs to bioaccumulate [5]–[7]. Indeed, PBDEs are lipophilic, extremely stable, and remain in products manufactured before the ban, as well as in new products made from recycled materials [1].

As PBDEs are lipophilic and accumulate in fat tissue, it is postulated that they may alter adipose tissue function and metabolism. Evidence supporting this hypothesis showed that adipocytes collected from rats exposed to daily doses of the DE-71 technical mixture exhibited increased lipolysis and decreased glucose oxidation, both hallmarks of obesity, insulin resistance, and type 2 diabetes [8]. Furthermore, a separate study showed that maternal exposure to the congener BDE-47 induced increases in body weights in offspring during early postnatal development [9]. Correlational studies in humans showed that there is a positive association between metabolic syndrome and serum levels of the congeners PBB-153 and PBDE-153 [10]. More recently, the presence of PBDEs and other persistent organic pollutants (POPs) in obese individuals was positively correlated with visceral fat and visceral/subcutaneous abdominal fat ratio [11]. Hence, the effects that PBDEs may have on adipose tissue function warrant further investigation.

In the current study, the 3T3-L1 cell model was used to study the process of preadipocyte differentiation in response to PBDEs. As a cocktail of cAMP enhancer, insulin, and glucocorticoid results in complete differentiation of preadipocytes, glucocorticoid was substituted with PBDEs in order to assess the potential of PBDEs to induce differentiation without high background. A mixture of PBDEs representative of body burden in North America was tested, along with the technical mixture DE-71, and the predominant congener in both of the mixtures, BDE-47. In addition, the potential for PBDEs to differentiate 3T3-L1 preadipocytes was tested in the presence of suboptimal concentrations of the synthetic glucocorticoid, dexamethasone. This study is the first to characterize the terminal differentiation of preadipocytes to mature adipocytes induced by these lipophilic contaminants.

## Materials and Methods

### Chemicals and reagents

Chemicals and reagents were obtained from the following manufacturers: DMEM/low glucose media and bovine calf serum (HyClone Laboratories, Thermo Scientific, Logan, Utah, U.S.A.); fetal bovine serum (Wisent Inc., Saint-Bruno, Quebec, Canada); human insulin (Roche Diagnostics, Indianapolis, Indiana, U.S.A.); 3-isobutyl-1-methylxanthine (IBMX), dexamethasone (DEX), and dimethyl sulfoxide (DMSO) (Sigma-Aldrich, Oakville, Ontario, Canada). PBDEs were obtained as follows: the technical mixture DE-71 was generously provided by Dr. Doug Arnold (Health Canada), from stock provided by Chemtura (Lawrenceville, Georgia, U.S.A.); DE-79 (Wellington Laboratories, Guelph, Ontario, Canada); purified BDE-47, BDE-28, BDE-100, and BDE-154 (AccuStandard Inc., New Haven, Connecticut, U.S.A.); purified BDE-209 (Sigma-Aldrich, Oakville, Ontario, Canada). Antibodies were purchased as follows: fatty acid binding protein 4 (aP2) (R&D Systems, Minneapolis, Minnesota, U.S.A); perilipin and β-actin (Cell Signaling Technology, Danvers, Massachusetts, U.S.A).

### Formulation of a PBDE mixture representative of body burden in North America

A literature review was conducted to determine the formulation of a mixture of PBDEs representative of body burden in North America. Our review was confined to papers that reported a broad range of congeners. Studies with breast milk provided the broadest range of PBDE congeners that were detected with >50% frequency and thus allowed the reliable determination of a median level of these among the populations studied. Three studies examining levels of PBDEs in breast milk from women in various locations across North America showed remarkable similarity in the relative proportions of individual congeners of total PBDE [12]–[14]. Therefore, the mixture was formulated to reflect the relative proportions of congeners observed in these studies (Table 1).

**Table 1:** Components of PBDE Mixture.

Component	%
DE–71	39.3
DE-79	0.7
BDE-209	1.0
BDE-47	40.8
BDE-100	5.0
BDE-28	3.9
BDE-153	9.3

### Cell culture

**Figure 1:**
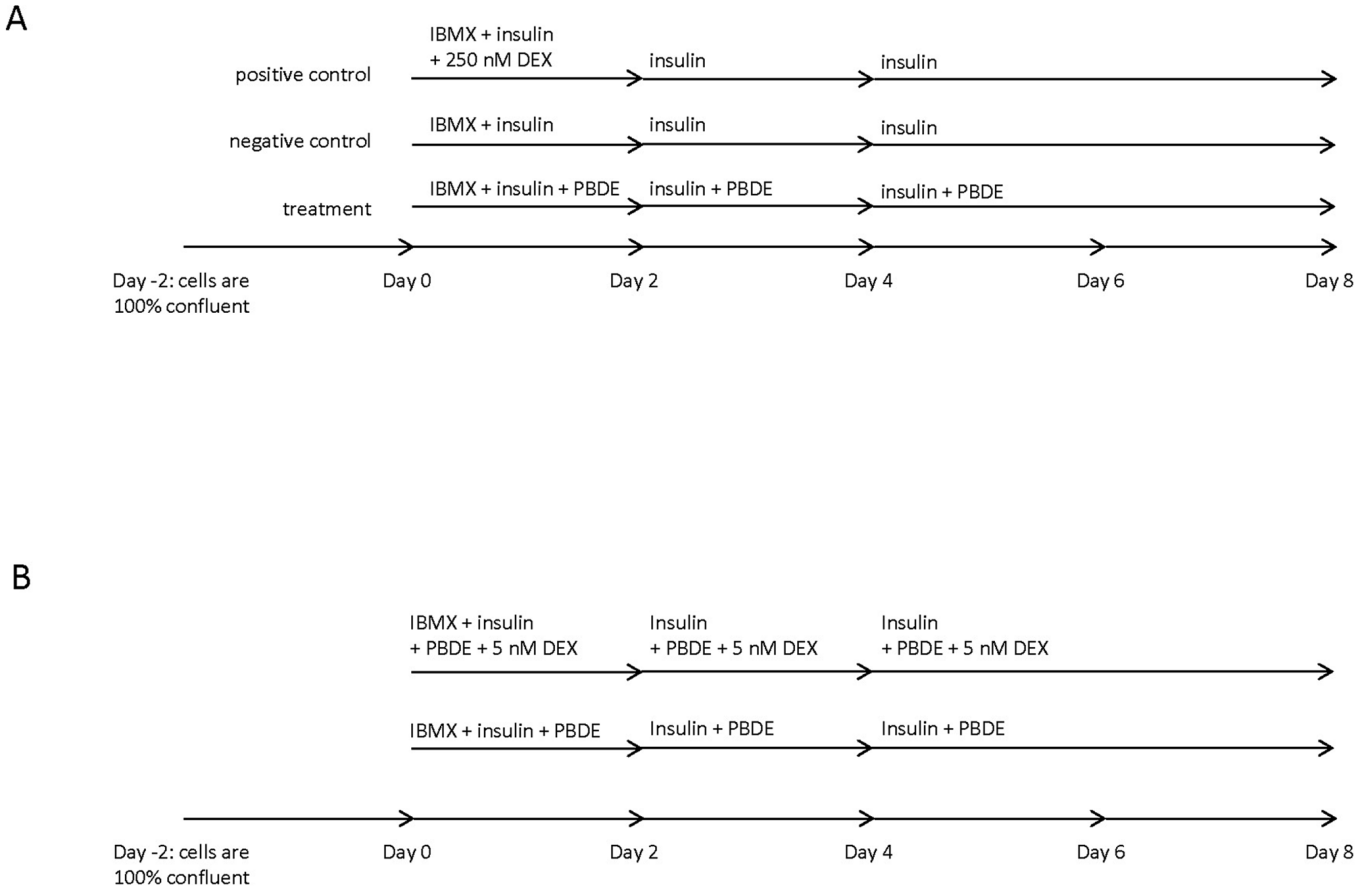
Exposure protocols for PBDEs. A) Differentiation protocol for PBDEs. B) Protocol for co-treatment with glucocorticoids (5 nM DEX).

3T3-L1 mouse embryo fibroblasts were cultured in DMEM containing 10% calf serum, maintained at confluence for 2 days, and induced to differentiate as illustrated in Figure 1. The differentiation cocktail consisted of 500 μM of the cAMP enhancer IBMX and 100 nM of insulin (MI), plus varying concentrations of PBDE mixture (DMSO, 1.6, 3.2, 6.4, 12.8, 25.5 μM), DE-71 (DMSO, 0.8, 1.5, 3, 6, or 12 μM), or BDE-47 (DMSO, 0.8, 1.5, 3, 6, or 12 μM) in DMEM containing 10% fetal bovine serum, 100 IU penicillin, and 100 μg/mL streptomycin.

### Cell viability

The MTT (3-[4,5-dimethylthiazol-2-yl]-2,5 diphenyl tetrazolium bromide) assay was conducted to determine the effect of PBDE treatment on cell viability. Cells were treated with PBDE mixture (3.2 and 25.5 μM), DE-71 (3 and 12 μM), and BDE-47 (1.5 and 12 μM) for 48 hours after which absorbance of dissolved formazan crystals was read at 590 nM.

### Western blotting of terminal differentiation markers

For Western blot analysis, cells were lysed in RIPA buffer (20 mM Tris pH 7.5, 150 mM NaCl, 1 mM EDTA, 1% sodium deoxycholate, 2% NP-40, 0.4% SDS, 10% glycine) containing protease inhibitors (Roche Diagnostics, Indianapolis, Indiana, U.S.A.). Protein were transferred to PVDF membranes and probed with antibodies against aP2, perilipin, or β-actin followed by HRP-linked secondary antibodies. Blots were developed using Clarity Western ECL Substrate (BioRad, Hercules, California, U.S.A.). Relative optical density was quantified using Image Lab software (BioRad). Protein loading was normalized using β-actin staining intensity.

### Immunofluorescence and quantification of adipocytes

Cells were seeded and differentiated in black, clear-bottomed 96 well plates. Cells were then fixed with 4% paraformaldehyde and stained with Nile red (1 μg/mL) to identify lipid droplets and DAPI (1 μg/mL) to stain nuclei. Nile red fluorescence was viewed at 530 nm and DAPI at 405 nm on a fluorescent microscope. Cells were photographed at 100× magnification. The number of mature adipocytes in the population was calculated as percentage of Nile red stained cells from the total number of cells. At least 300 cells were counted per well.

### Transfections and luciferase activity assay

3T3-L1 cells were transfected in triplicate with FuGENE 6 (Roche Applied Science), as per manufacturer's instructions, using 20 ng of pTL-GR expression vector [15], 100 ng of -237 MMTV-luciferase reporter [16], and 20 ng of pRL-CMV as an internal control (Promega Corporation, Madison, Wisconsin, U.S.A.). Cells were treated with PBDE mixture (25.5 μM), DE-71 (12 μM), and BDE-47 (12 μM), or DEX (10 nM) for 48 hours, lysed, and evaluated for luminescence. Activity was normalized to the CMV internal control.

### Real-time PCR

Cells were harvested on days 1, 2, 4, 5, 6, and 8. RNA was extracted using the RNeasy Mini kit (Qiagen, Missisauga, Canada) and then reverse transcribed using iScript cDNA Synthesis Kit (BioRad). The expressions of the following genes were determined using SsoFast EvaGreen supermix: aP2, perilipin, CCAAT-enhancer binding protein α (C/EBPα), peroxisome proliferator-activated receptor γ (PPARγ), and liver X receptor α (LXRα) (Table S1). PCR efficiency was examined using standard curves for each gene using pooled cDNA from all control samples. Relative gene expression was normalized to β-actin gene expression.

### Statistical analyses

Statistical analyses were performed with SigmaPlot 12.5 software. One way analysis of variance was conducted, followed by Holm-Sidak post-hoc test. For non-normal data, a Kruskal-Wallis one way ANOVA on ranks was performed, followed by Dunnett's post-hoc test. For dexamethasone co-treatment, two way ANOVA was performed followed by Holm-Sidak post-hoc test to compare all groups. When normality failed, the natural logarithm of each data value was determined. Data are presented as mean ± SEM.

## Results

### Cell viability

Treatment of undifferentiated 3T3-L1s with the PBDE mixture, DE-71, or BDE-47 did not induce cytotoxicity as indicated by MTT assay (Figure S1). Moreover, no evidence of cytotoxicity was observed in any treated cultures during the 8 days of differentiation.

### PBDE exposure induced protein and mRNA expression of adipogenic markers

**Figure 2:**
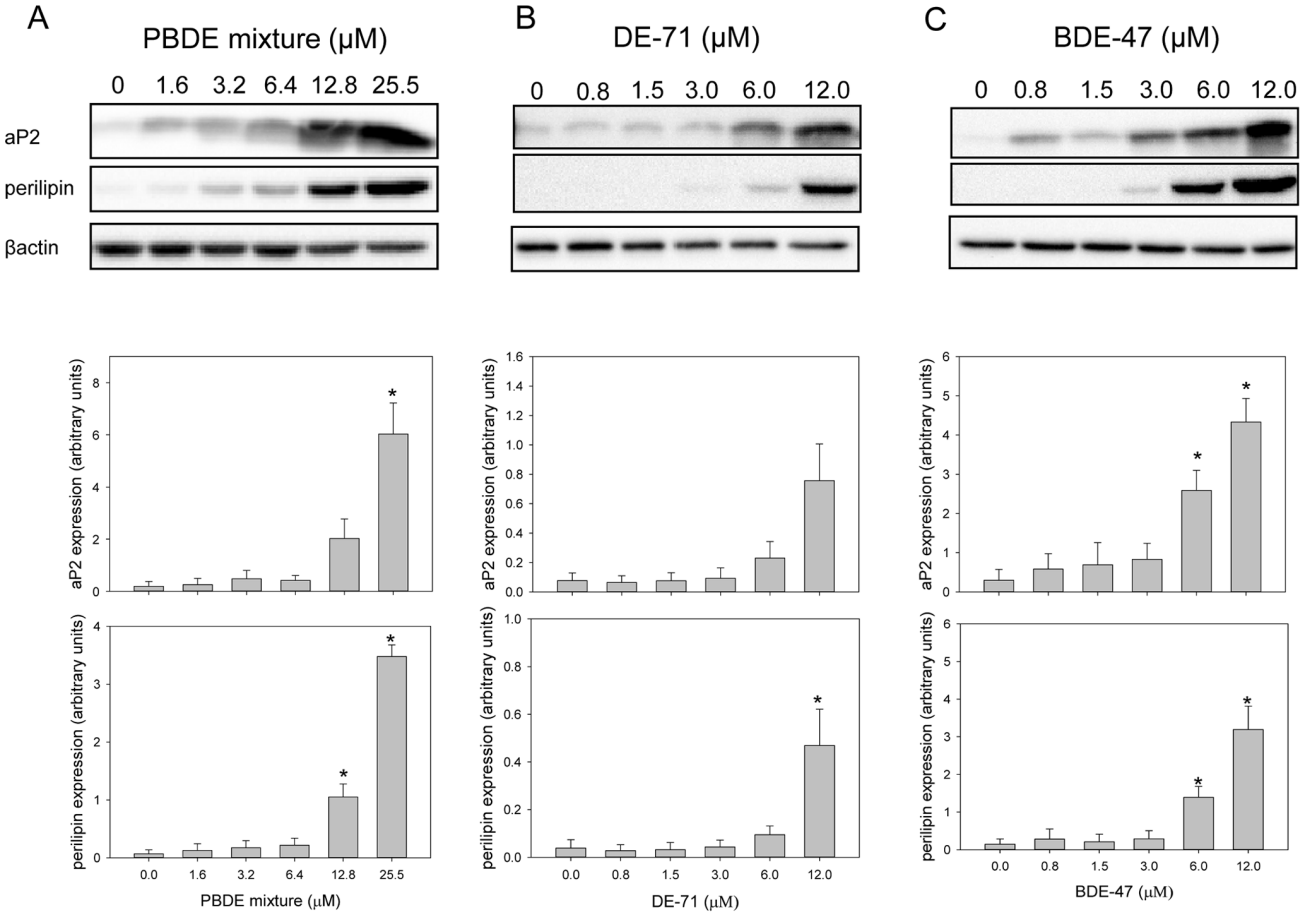
Western blotting for terminal differentiation markers. A) PBDE-induced changes in aP2 and perilipin protein expression, presented with representative blots. Data is expressed as mean ± SEM, n = 3 independent experiments. B) Relative protein expression of aP2 and perilipin following exposure to DE-71, presented with representative blots. Data is expressed as mean ± SEM, n = 4 independent experiments. C) BDE-47-induced changes in aP2 and perilipin expression, presented with representative blots. Data is expressed as mean ± SEM, n = 4 independent experiments. * indicates statistical significance from control, p<0.05.

**Figure 3:**
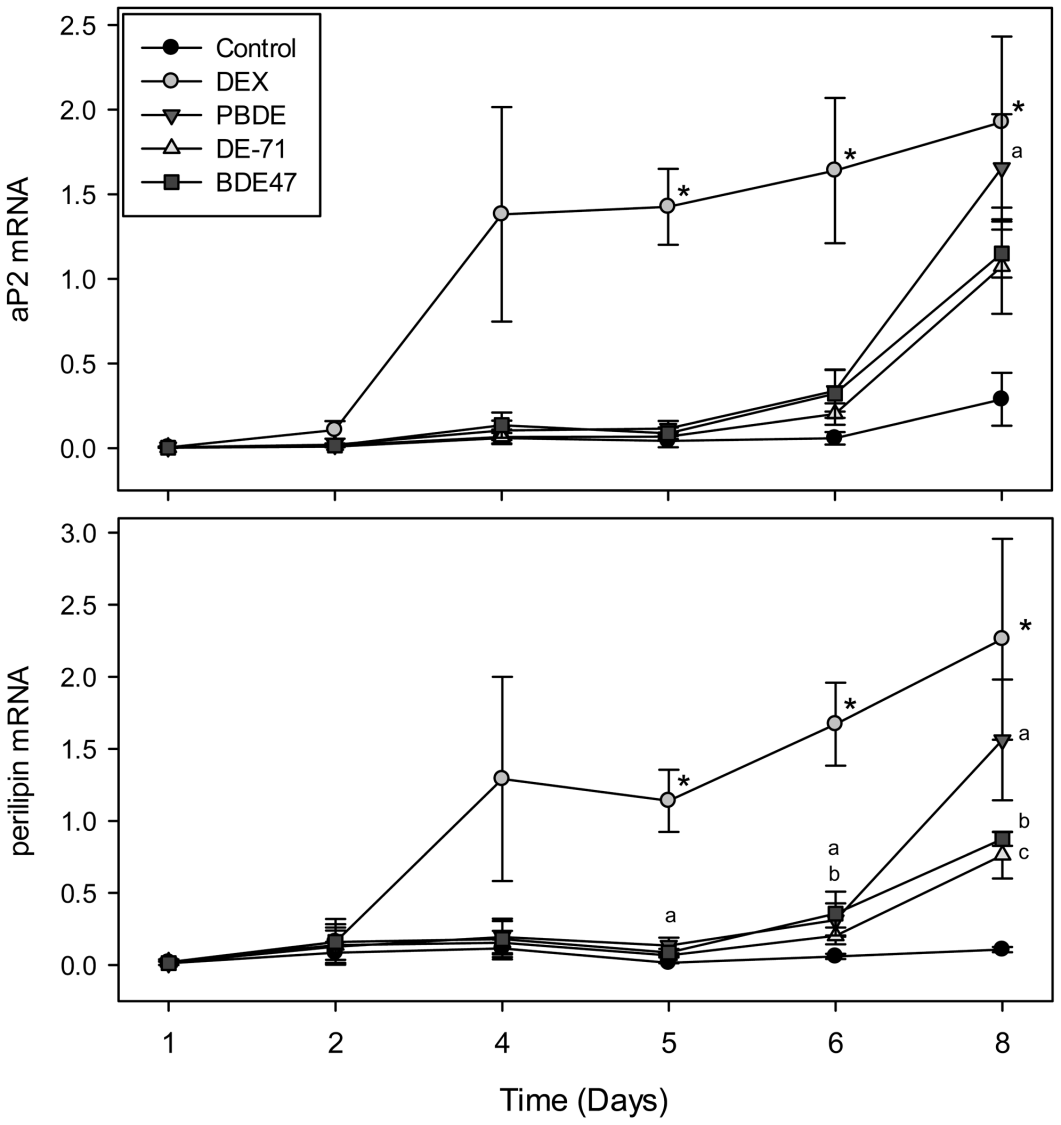
RT-PCR for terminal differentiation markers expressed in relative units following treatment with 250 nM DEX, 25.5 μ**M PBDE mixture, 12** μ**M DE-71, or 12** μ**M BDE-47.** With regards to aP2 RNA expression levels, for days 1, 2, 4, the number of experimental replicates is 4 for all groups except 250 nM DEX (n = 3). For days 5, 6, 8, the number of experimental replicates is 5 for all treatment groups. With regards to perilipin RNA expression levels, for days 1, 2, 4, the number of experimental replicates is 3 for all treatment groups. For days 5, 6, 8, the number of experimental replicates is 5 for all treatment groups. * indicates a significant difference between control and DEX within the given time point (p<0.05). ^a^ indicates a significant difference between control and PBDE (p<0.05). ^b^ indicates a significant difference between control and BDE-47 (p<0.05). ^c^ indicates a significant difference between control and DE-71 (p<0.05). Data is expressed as mean ± SEM.

To assess the potential for PBDEs to induce differentiation of 3T3-L1 preadipocytes, cells were treated with a mixture of PBDEs representative of body burden in North America, the technical mixture DE-71, and the congener BDE-47. The extent of differentiation was assessed by measurement of protein and transcript levels of adipogenic markers from all cells collected from the culture well at specific time points to give average expression changes across the cellular mixture consisting of fibroblasts and adipocytes. Treatment of the 3T3-L1 cells with PBDEs using the regiment illustrated in Figure 1A resulted in significant increases in aP2 (at 25.5 μM) and perilipin (at 12.8 and 25.5 μM) protein levels (Figure 2A). The technical mixture DE-71 induced a dose dependent increase in perilipin expression which reached significance at 12 μM, and an increasing trend was observed for aP2 (Figure 2B). Treatment with 6 or 12 μM BDE-47 induced significant, dose dependent increases in aP2 and perilipin (Figure 2C). The positive control of 250 nM DEX induced robust increases in aP2 and perilipin expression (40-fold and 97-fold over control, respectively) (data not shown). aP2 and perilipin RNA expression was measured throughout the differentiation process (Figure 3). 250 nM DEX increased aP2 and perilipin expression levels significantly from controls (DMSO) at days 5, 6, and 8. Perilipin mRNA levels were significantly increased by the PBDE mixture at days 5, 6, and 8 of differentiation, reaching a 15-fold increase at day 8. aP2 mRNA expression levels were also significantly increased by PBDE treatment at day 8 compared to controls (6-fold). The congener BDE-47 (12 μM) significantly increased perilipin expression at days 6 and 8 of differentiation reaching 8-fold over MI controls, and DE-71 (12 μM) significantly increased perilipin mRNA levels at day 8 (7-fold).

### PBDE exposure increases staining of lipids

**Figure 4:**
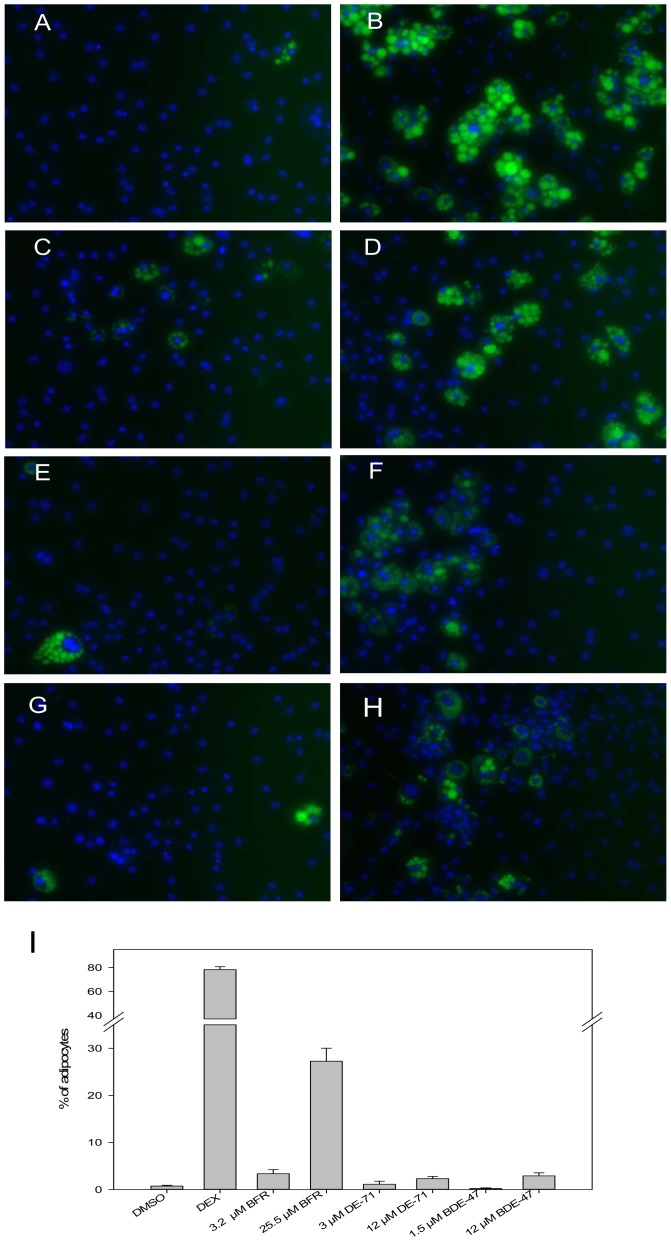
Nile red staining of lipids. A) DMSO. B) 250 nM DEX. C) 3.2 μM PBDE mixture. D) 25.5 μM PBDE mixture. E) 3 μM DE-71. F) 12 μM DE-71. G) 1.5 μM BDE-47. H) 12 μM BDE-47. I) Percentage of adipocytes present following differentiation.

Nile red staining was performed to visualize lipid accumulation on day 8 of differentiation. Increased lipid accumulation was observed with DEX, 25.5 μM PBDE mixture, 12 μM DE-71, and 12 μM BDE-47 on day 8 (Figure 4). Adipocyte counts were significantly increased following exposure to DEX and PBDE mixture (78% for DEX and 27% for PBDEs). A modest increase was also observed in DE-71 and BDE-47 treated cultures.

### PBDEs do not affect glucocorticoid receptor-mediated transcription on the MMTV promoter

To test the ability of PBDEs to induce MMTV promoter activity via glucocorticoid receptor (GR), luciferase assays were performed. Treatment of transfected 3T3-L1 cells with PBDEs (25.5 μM), DE-71 (12 μM), or BDE-47 (12 μM) did not affect MMTV promoter activity in the absence or presence of DEX (10 nM) (Figure S2).

### Glucocorticoids enhance PBDE-induced adipocyte differentiation

To assess PBDE interaction with glucocorticoids, 3T3-L1 cells were differentiated in the presence of a low dose of DEX (5 nM). The results are summarized in Table 2. Treatment with 5 nM DEX alone resulted in a 7-fold increase in aP2 expression and 15-fold increase in perilipin expression when compared to the MI control. When the preadipocytes were treated with 3.2 μM PBDE mixture and DEX, perilipin expression was further increased by 4-fold compared to 3.2 μM PBDE mixture alone, and enhanced DEX-mediated increases by 1.4-fold. BDE-47 co-treatment with DEX enhanced aP2 expression by 3-fold compared to 12 μM BDE-47 alone and 4-fold compared to DEX, although these changes did not reach statistical significance. Both 1.5 μM and 12 μM BDE-47 with DEX co-treatment induced significant increases in perilipin expression compared to 1.5 and 12 μM BDE-47 alone, and induced 2-fold and 5-fold increases over DEX alone. 12 μM DE-71 co-treatment with DEX enhanced DEX-mediated aP2 and perilipin expression by 4.5-fold and 4-fold, respectively.

**Table 2:** PBDE interaction with glucocorticoids.

	aP2 protein expression (arbitrary units)		
Treatment	+ 0 nM DEX	+ 5 nM DEX	P (0 nM vs. 5 nM DEX)
DMSO	0.11±0.09 (3)	0.77±0.41 (3)	0.002
3.2 μM PBDE mixture	0.29±0.17 (3)	1.21±0.76 (3)	0.09
25.5 μM PBDE mixture	1.23±0.22* (3)	5.09±0.74 (3)	0.11
3 μM DE-71	0.26±0.16 (3)	1.17±0.75 (3)	0.14
12 μM DE-71	0.81±0.50 (3)	3.48±1.81 (3)	0.054
1.5 μM BDE-47	0.07±0.04 (3)	1.17±0.30 (3)	<0.001
12 μM BDE-47	1.01±0.11* (3)	3.41±0.28 (3)	0.17

	perilipin protein expression (arbitrary units)		
Treatment	+ 0 nM DEX	+ 5 nM DEX	P (0 nM vs. 5 nM DEX)
DMSO	0.03±0.04 (3)	0.45±0.11 (3)	0.002
3.2 μM PBDE mixture	0.15±0.14 (3)	0.64±0.45 (3)	0.03
25.5 μM PBDE mixture	0.67±0.15* (3)	2.67±0.54 (3)	0.13
3 μM DE-71	0.09±0.06 (3)	0.63±0.44 (3)	0.054
12 μM DE-71	0.35±0.19 (3)	1.70±0.63 (3)	0.054
1.5 μM BDE-47	0.07±0.02 (3)	0.79±0.27 (3)	0.01
12 μM BDE-47	0.62±0.30 (3)	2.33±0.16 (3)	0.03

Data is expressed as mean ± SEM of 3 independent experiments for all treatment groups. This data set was ln-tranformed prior to analysis. * indicates a significant difference from DMSO/0 nM DEX control, p<0.03. P values indicating a significant difference between treatment with PBDEs alone versus PBDEs with 5 nM DEX are indicated in the table.

### PBDE exposure induces RNA expression of transcription factors and nuclear receptors required for adipogenesis

**Figure 5:**
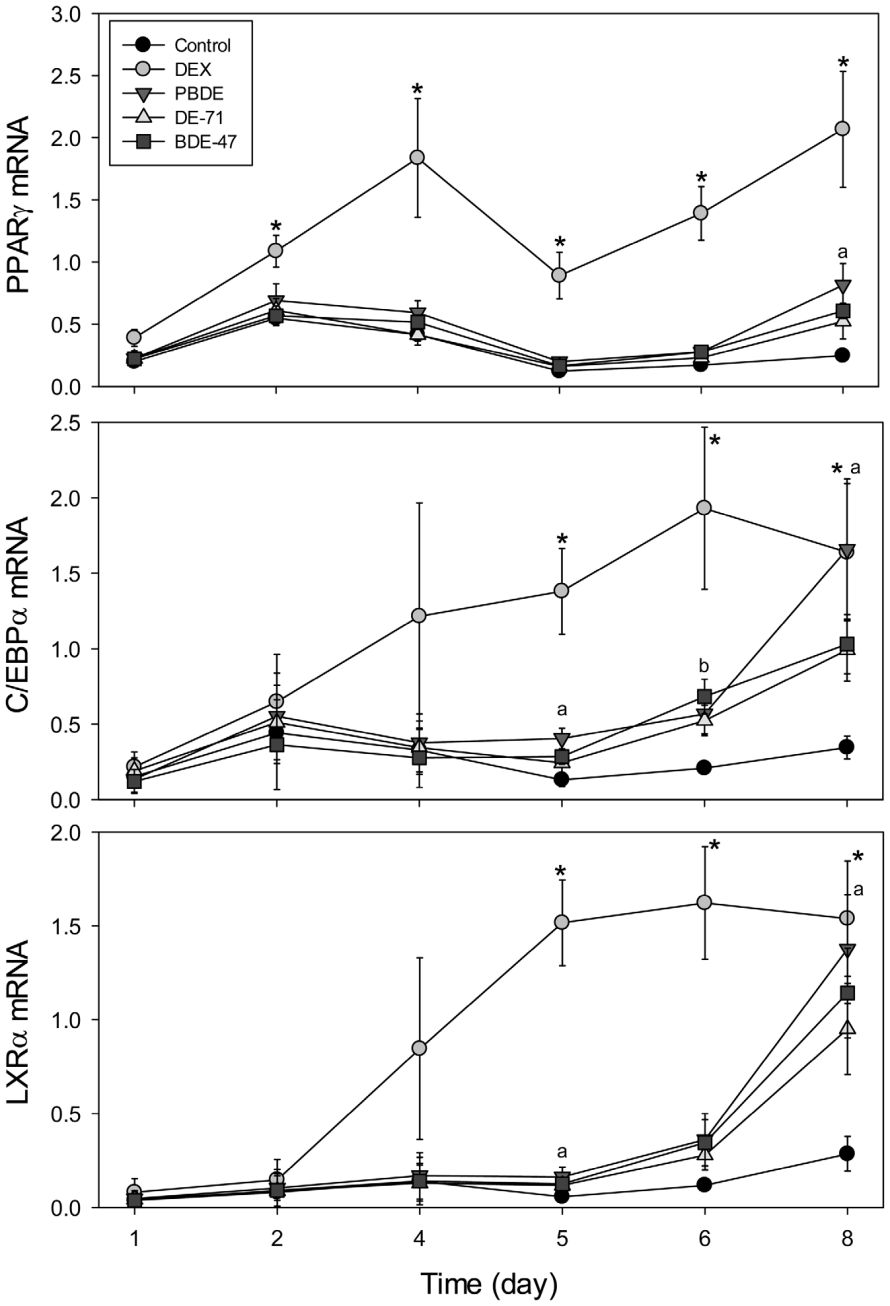
mRNA Expression of PPARγ, C/EBPα, and LXRα during differentiation following treatment with 250 nM DEX, 25.5 μ**M PBDE, 12** μ**M DE-71, or 12** μ**M BDE-47 in relative units.** For *PPARγ* RNA expression during days 1, 2, 4, the number of experimental repeats is 4 for all treatment groups. For days 5, 6, 8, the number of experimental repeats is 5 for all treatment groups. For *C/EBPα* and LXRα RNA expression during days 1, 2, 4, the number of experimental repeats is 3 for all treatment groups. For days 5, 6, 8, the number of experimental repeats is 5 for all treatment groups. * statistical difference between control and DEX at the given time point, p<0.05; ^a^ statistical difference between control and PBDE, p<0.05; ^b^ statistical difference between control and BDE-47, p<0.05. Data is expressed as mean ± SEM.

To characterize the transcriptional cascade involved in PBDE-induced differentiation, *PPARγ, C/EBPα, and LXRα* mRNA levels were determined by RT-PCR following exposure to the highest doses of flame retardants tested (Figure 5). *PPARγ* mRNA levels were induced by DEX beginning at day 2, and this increase was sustained throughout the differentiation process. Treatment with the PBDE mixture, DE-71, or BDE-47 throughout the differentiation process caused increases in *PPARγ* transcript levels at day 8, although only the treatment with the PBDE mixture reached statistical significance.

*C/EBPα* RNA expression was increased by DEX as early as day 4. The PBDE mixture induced a slight increase in *C/EBPα* levels at day 5, although a more marked increase was evident only at day 8. DE-71 and BDE-47 increased *C/EBPα* RNA levels starting at day 5; however, these changes were more modest.

As expected, *LXRα* RNA expression was increased by DEX significantly at days 5, 6, and 8. The PBDE mixture (25.5 μM) induced significant increases in *LXRα* RNA levels as early as day 5, which was maintained until day 8. DE-71 and BDE-47 increased *LXRα* transcript levels at days 6 and 8, although these changes were not statistically significant.

## Discussion

In the current study, we show that a PBDE mixture, DE-71, and BDE-47 induce an increase in aP2 and perilipin expression in the mixture of preadipocytes and mature adipocytes at the end of the differentiation process. Cell counts of the cells that stained positively with Nile red, a dye specific for lipid, show a significant increase in adipocyte numbers following treatment with the PBDE mixture, more so than with DE-71 or BDE-47. As the content of BDE-47 in the PBDE mixture is comparable to the highest dose of BDE-47 tested, it is possible that the components of the mixture may be acting together to enhance the adipogenic effect. In humans, the most prevalent congeners found in fat depots are BDE-47, BDE-99, and BDE-153 [17]. Hence, future studies directed towards testing the other congeners on their own and in combination would provide valuable, environmentally-relevant data on PBDE-induced adipogenesis.

Although current literature regarding the adipogenic effects of PBDEs is limited, a recently published report tested the differentiation potential of BDE-47 by exposing 3T3-L1 pre-adipocytes to IBMX, insulin, and DEX (MID), in addition to BDE-47 [18]. These experiments showed approximately a 1.5-fold increase in differentiation with 12 μM BDE-47 compared to controls exposed to MID. In our system, we omitted DEX when testing the differentiation potential of the PBDEs in order to delineate the mechanism of action. It is important to note that in the absence of DEX, 3T3-L1 cells do not undergo significant differentiation. Therefore, it is particularly striking that in the absence of DEX, PBDEs induce the differentiation process in 3T3-L1s. Although these results suggest that PBDEs may be acting as glucocorticoids, neither the PBDE mixture, DE-71, or BDE-47 could induce glucocorticoid-mediated MMTV-luciferase activity in the presence or absence of DEX. This suggests that the PBDEs do not act on GR-mediated transcriptional activity, or at least they do not influence GR action on the MMTV promoter. It is also intriguing that in our experiments, co-treatment of the cells with the PBDEs and DEX resulted in an additive effect which further suggests that the PBDEs act on a different molecular target than the glucocorticoid receptor. This may have implications on the potency of the flame retardants in conditions where higher glucocorticoids are present, such as under stress [19].

The effects of PBDEs on the expression levels of key transcription factors were tested by measuring mRNA expression. Compared to DEX, exposure to the PBDE mixture, DE-71, or BDE-47 resulted in the late induction of PPARγ and C/EBPα on days 5-8, which in turn was sufficient to induce the adipogenic factor, LXRα [20]–[22]. It has been postulated that LXRα regulates lipogenesis and maintenance of the mature adipocyte phenotype, but not adipogenesis itself [23]. However, the role of this transcription factor in adipogenesis is controversial and it may be involved in PPARγ regulation. A previous study demonstrated that treatment of the 3T3-L1s with an LXRα agonist induces the expression of PPARγ [21]. As PPARγ can also upregulate LXRα expression, these two transcription factors may form a positive-feedback loop that then pushes the adipocyte differentiation process forward [21], [24]. It is therefore possible that the PBDEs are acting through LXRα or PPARγ.

The 3T3-L1 cell line is widely used as a model to study adipogenesis, and has been used to establish most of the molecular events that occur in the differentiation process; however, there are limitations to this model [25]. This cell line may not be completely reflective of adipogenesis in human preadipocytes as it was derived from clonal expansion of isolated rodent fibroblasts, and may not accurately represent the variations in micro-environments observed in different fat depots *in vivo* [26]. As observed in many other cell lines, the 3T3-L1s do not appear to have the capability for metabolism of some xenobiotics [27]. Differences may also exist between the delivery of PBDEs to the target tissue *in vivo* compared to cell culture, although the precise mechanism in both models is unknown. *In vivo*, PBDEs are thought to circulate by binding to serum proteins, as well as serum lipoproteins [28]–[30]. As 96% of PBDEs administered in culture remains serum-bound, PBDEs are possibly binding to proteins and/or lipoproteins present in the serum [30], [31]. In addition, uptake of PBDEs by active transporters has been reported; however, it is unknown whether these are present in 3T3-L1 fibroblasts [32]. While the precise mechanisms by which delivery and uptake occur remain unknown *in vitro* and *in vivo*, different mechanisms may be involved, and that may result in comparatively different adipogenic responses. Nevertheless, the 3T3-L1 preadipocyte model remains a useful tool for identification of adipogenic agents, as well as characterization the molecular pathways involved in adipogenesis. Here, we identified PBDEs as a potential adipogenic agent in the 3T3-L1 cell line and although these results were obtained *in vitro*, the molecular targets may be relevant *in vivo*.

Previously reported values regarding human body burden of PBDEs vary regionally. Highest levels were observed in occupational exposures, specifically at electronics dismantling plants, with serum values ranging from 3.8-8500 ng/g lipid for total PBDEs [33]. Assuming that the lipid content of serum is approximately 0.3–0.5%, the lowest dose of BDE-47 that induced an effect on aP2 or perilipin expression in the current study (1.5 μM) represents a concentration that is approximately 20-fold higher than the highest serum levels for total PBDEs measured in occupational exposures. While the nominal concentrations used in the current study are higher than what would be expected in serum samples from the general population, the actual concentration of our test substance at the cellular level in the current *in vitro* model is not clear. Transfer of BDE-47 from culture media to cells *in vitro* was previously shown to be inhibited by the addition of serum in the media, as only 3% of the applied PBDE was associated with the cells following an hour of incubation, and 96% remained in the media [30]. In addition, as PBDEs tend to accumulate in adipose depots, it is possible that human preadipocytes may be exposed to higher concentrations of PBDEs than found in serum due to the physiological proximity of these cells to white adipose cells. While the ranges of concentrations used in this study are higher than reported serum concentrations, their effects on adipogenesis may be physiologically relevant due to the inherent properties of cell culture, as well as bioaccumulative effects *in vivo*.

In this study, we present the novel finding that a PBDE mixture designed to mimic body burden in North America, the technical mixture DE-71, and the congener BDE-47 induces differentiation of 3T3-L1 preadipocytes in the absence of glucocorticoid. Examination of the transcriptional cascade indicated that C/EBPα and PPARγ are upregulated late in the differentiation process by the PBDEs, suggesting that the mechanism of action may be different from that of glucocorticoid. Future studies are required to examine these mechanisms, as the lipophilic and bioaccumulative nature of these chemicals warrant further examination on the effects of adipogenesis and health.

## Supporting Information

Figure S1 Cell viability as assessed by MTT assay. Data is expressed as mean ± SEM, n = 3. Experiments were performed in triplicate.
(TIFF)

Figure S2 A. Effect of PBDEs on MMTV promoter activity. Data is expressed as mean ± SEM, n = 3. B. Effect of PBDEs on MMTV promoter activity in the presence of 10 nM DEX. Data is expressed as mean ± SEM, n = 2. Experiments were performed in triplicate.
(TIF)

Table S1 **Primer sequences for real-time PCR.**
(TIFF)
