# Supplementary figures and images for: Induction of Adipocyte Differentiation by Polybrominated Diphenyl Ethers (PBDEs) in 3T3-L1 Cells
Source: PLoS One. 2014 Apr 10;9(4):e94583. doi: 10.1371/journal.pone.0094583 (PMC3983240; doi:10.1371/journal.pone.0094583)

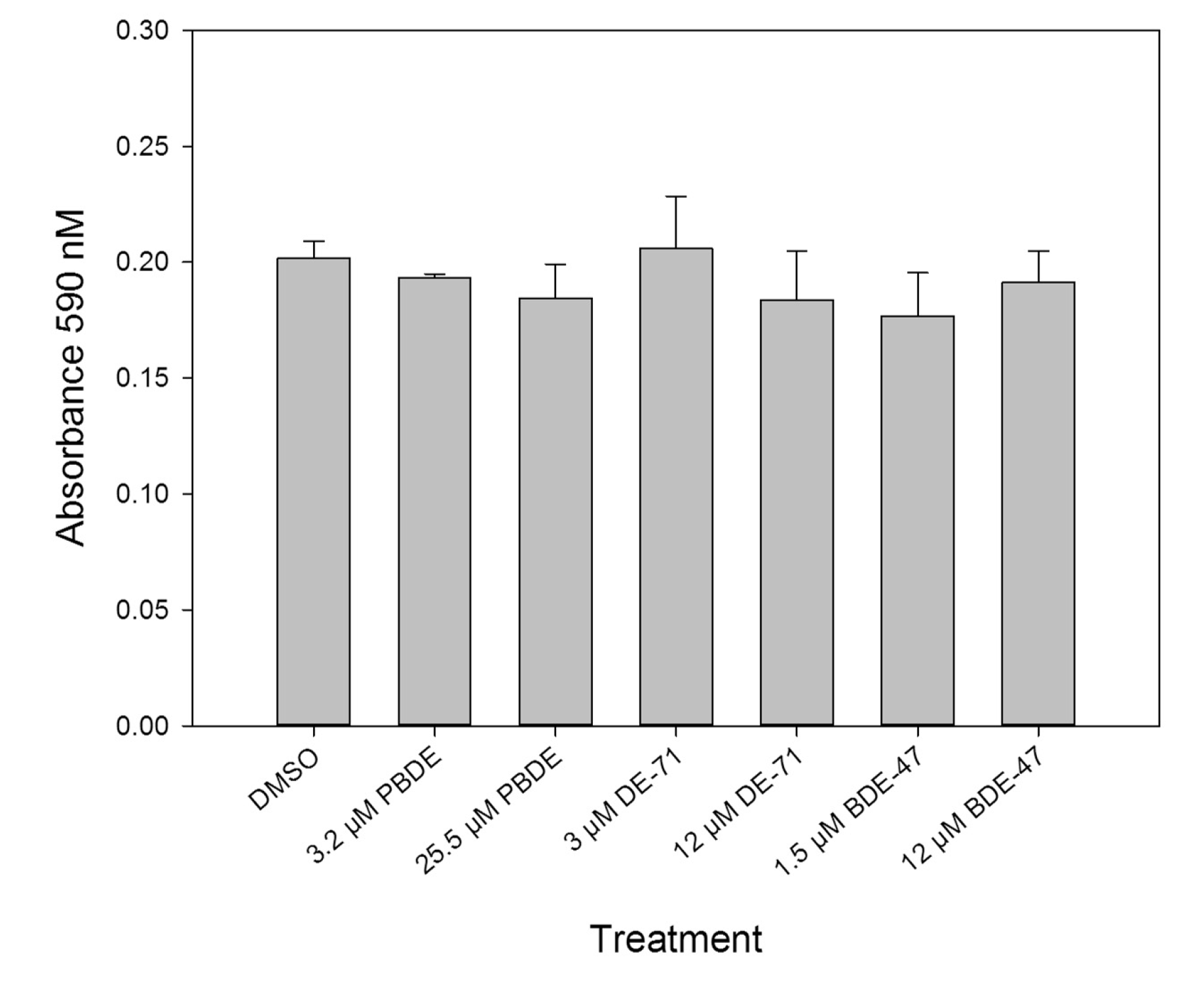

Supplement: Figure S1 — Cell viability as assessed by MTT assay. Data is expressed as mean ± SEM, n = 3. Experiments were performed in triplicate. (TIFF) [file pone.0094583.s001.tif]

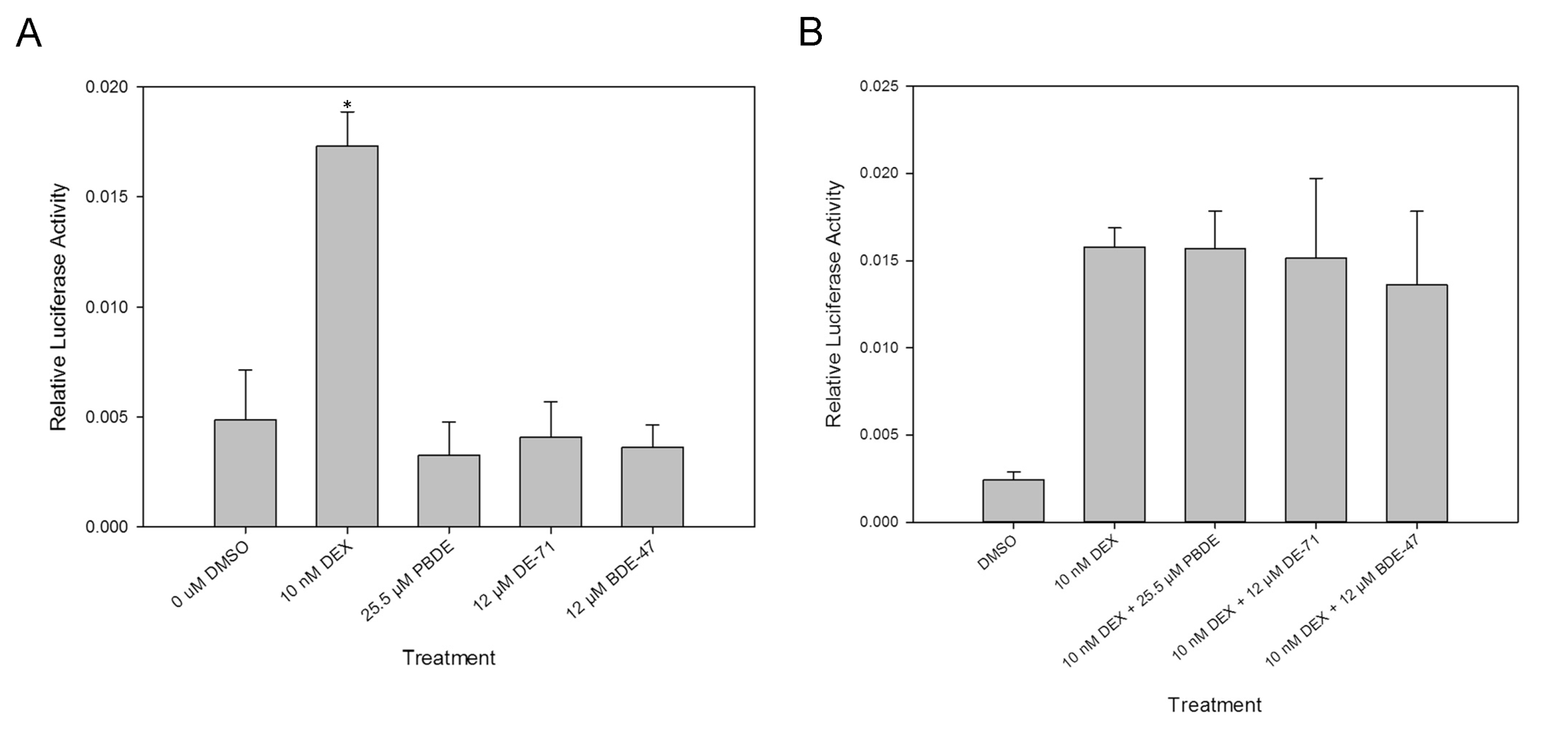

Supplement: Figure S2 — A. Effect of PBDEs on MMTV promoter activity. Data is expressed as mean ± SEM, n = 3. B. Effect of PBDEs on MMTV promoter activity in the presence of 10 nM DEX. Data is expressed as mean ± SEM, n = 2. Experiments were performed in triplicate. (TIF) [file pone.0094583.s002.tif]

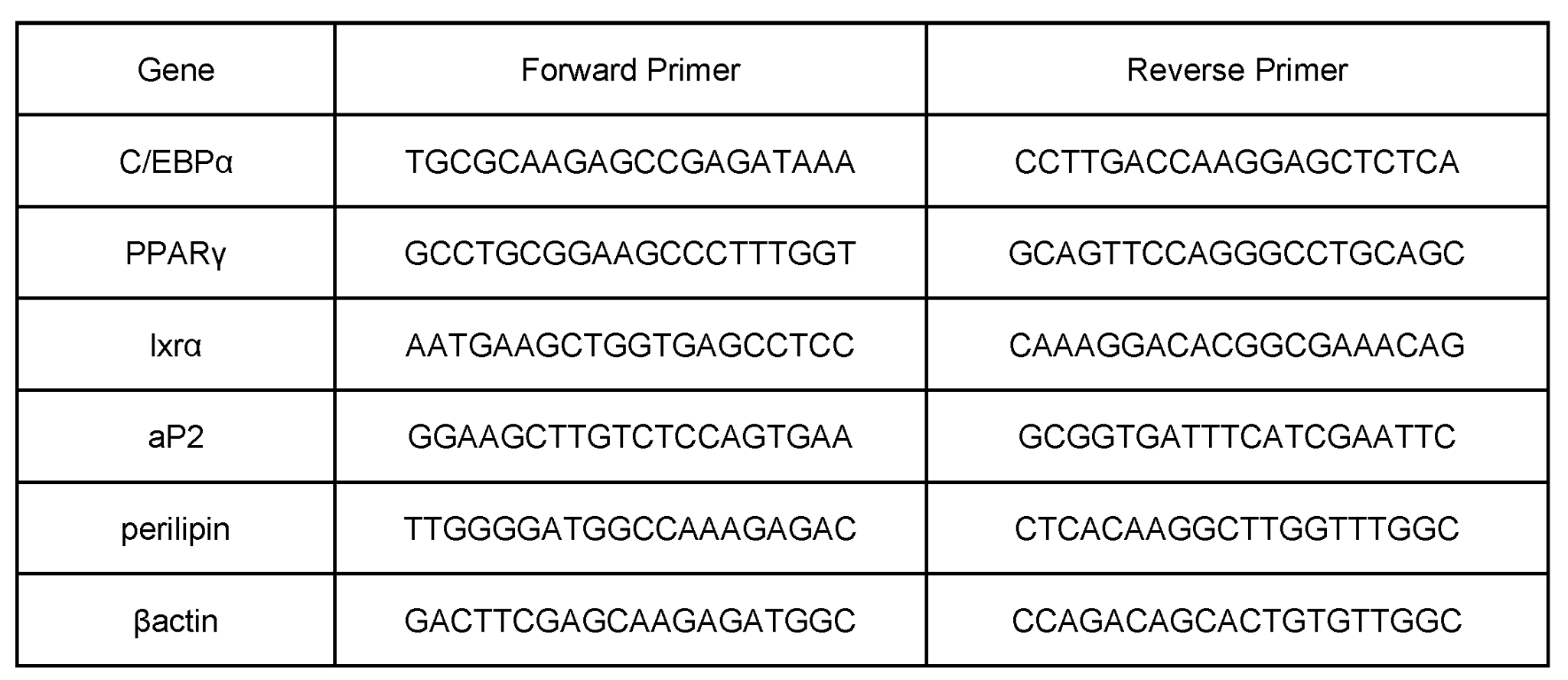

Supplement: Table S1 — Primer sequences for real-time PCR. (TIFF) [file pone.0094583.s003.tif]
